# Maintaining Implementation through Dynamic Adaptations (MIDAS): protocol for a cluster-randomized trial of implementation strategies to optimize and sustain use of evidence-based practices in Veteran Health Administration (VHA) patients

**DOI:** 10.1186/s43058-022-00297-z

**Published:** 2022-05-14

**Authors:** Laura J. Damschroder, Jeremy B. Sussman, Paul N. Pfeiffer, Jacob E. Kurlander, Michelle B. Freitag, Claire H. Robinson, Patrick Spoutz, Melissa L.D. Christopher, Saraswathy Battar, Kimberly Dickerson, Christopher Sedgwick, Ashleigh G. Wallace-Lacey, Geoffrey D. Barnes, Amy M. Linsky, Christi S. Ulmer, Julie C. Lowery

**Affiliations:** 1 Center for Clinical Management Research, VA Ann Arbor Healthcare System, 2215 Fuller Road, Ann Arbor, MI 48105 USA; 2 Department of Internal Medicine, University of Michigan, Ann Arbor, MI USA; 3 Institute for Healthcare Policy and Innovation, Ann Arbor, MI USA; 4 Department of Psychiatry, University of Michigan, Ann Arbor, MI USA; 5 Veterans Health Affairs VISN 20 Pharmacy Benefits Management, Vancouver, WA USA; 6 Pharmacy Benefits Management Services, Veterans Health Administration, 810 Vermont Ave NW, Washington DC, 20420 USA; 7 Michael E. DeBakey Veterans Affairs Medical Center, Houston, TX USA; 8 Baylor College of Medicine, Houston, TX USA; 9 Central Arkansas Veterans Healthcare System, Little Rock, AR USA; 10 Department of Veterans Affairs, VA Heartland Network (VISN 15), Kansas City, MO USA; 11 VA St Louis Medical Center John Cochran Division, St. Louis, MO USA; 12 Frankel Cardiovascular Center, University of Michigan, Ann Arbor, MI USA; 13 Section of General Medicine, VA Boston Healthcare System, Boston, MA USA; 14 Center for Health Organizations and Implementation Research, VA Boston Healthcare System, Boston, MA USA; 15 Section of General Internal Medicine, Boston University School of Medicine, Boston, MA USA; 16 Center of Innovation to Accelerate Discovery and Practice Transformation (ADAPT) Durham Veterans Affairs Health Care System, Durham, NC USA; 17 Department of Psychiatry and Behavioral Sciences, Duke University, Durham, NC USA

**Keywords:** Implementation science, Medication safety, Deprescribing, Polypharmacy, Insomnia, Anti-coagulation, Academic detailing, Implementation strategy, Quality improvement

## Abstract

**Background:**

The adoption and sustainment of evidence-based practices (EBPs) is a challenge within many healthcare systems, especially in settings that have already strived but failed to achieve longer-term goals. The Veterans Affairs (VA) Maintaining Implementation through Dynamic Adaptations (MIDAS) Quality Enhancement Research Initiative (QUERI) program was funded as a series of trials to test multi-component implementation strategies to sustain optimal use of three EBPs: (1) a deprescribing approach intended to reduce potentially inappropriate polypharmacy; (2) appropriate dosing and drug selection of direct oral anticoagulants (DOACs); and (3) use of cognitive behavioral therapy as first-line treatment for insomnia before pharmacologic treatment. We describe the design and methods for a harmonized series of cluster-randomized control trials comparing two implementation strategies.

**Methods:**

For each trial, we will recruit 8–12 clinics (24–36 total). All will have access to relevant clinical data to identify patients who may benefit from the target EBP at that clinic and provider. For each trial, clinics will be randomized to one of two implementation strategies to improve the use of the EBPs: (1) individual-level academic detailing (AD) or (2) AD plus the team-based Learn. Engage. Act. Process. (LEAP) quality improvement (QI) learning program. The primary outcomes will be operationalized across the three trials as a patient-level dichotomous response (yes/no) indicating patients with potentially inappropriate medications (PIMs) among those who may benefit from the EBP. This outcome will be computed using month-by-month administrative data. Primary comparison between the two implementation strategies will be analyzed using generalized estimating equations (GEE) with clinic-level monthly (13 to 36 months) percent of PIMs as the dependent variable. Primary comparative endpoint will be at 18 months post-baseline. Each trial will also be analyzed independently.

**Discussion:**

MIDAS QUERI trials will focus on fostering sustained use of EBPs that previously had targeted but incomplete implementation. Our implementation approaches are designed to engage frontline clinicians in a dynamic optimization process that integrates the use of actional clinical data and making incremental changes, designed to be feasible within busy clinical settings.

**Trial registration:**

ClinicalTrials.gov: NCT05065502. Registered October 4, 2021—retrospectively registered.

**Supplementary Information:**

The online version contains supplementary material available at 10.1186/s43058-022-00297-z.

Contributions to the literature

- A uniquely designed series of cluster-randomized controlled trials that assess a unified primary outcome across three evidence-based practices
- Protocol for testing two implementation approaches that target teams versus individual clinicians within clinical settings
- Focus on sustained outcomes based on continued involvement of individuals and/or teams in optimizing clinical processes

## Background

Sustaining the use of evidence-based practices (EBPs) is a well-documented challenge for health systems [1, 2]. In the Department of Veterans Affairs (VA), Quality Enhancement Research Initiative (QUERI) programs have long worked to close the gap between evidence and practice, focusing on implementing sustained routine use of EBPs [3]. As part of a recently funded QUERI program, the Maintaining Implementation through Dynamic Adaptations (MIDAS) program aims to directly improve care for Veterans by engaging frontline clinicians to not only optimize care by closing documented quality gaps but also to *sustain* those improvements. Too often, initial implementation is “spotlighted” through high attention and leadership priority, but many clinics struggle to achieve and/or sustain positive impact after the spotlight turns to other initiatives [4]. Lack of sustainment may be particularly challenging for clinics that start off with low rates of EBP use compared to other clinics in the system [5]. The problem of implementation sustainability is due, in part, to the fact that many implementation efforts are short in duration with limited follow-up support [6, 7]. Strategies that change the habits of practitioners—either by changing the system within which they practice or by providing insights about how to make doing the EBP simpler and more meaningful—are likely to be more effective [8].

We will conduct a series of trials that each aims to implement an EBP with documented quality gaps. Clinics will be randomized to one of two implementation strategies designed to make *sustained* changes [4]; patient-level use of potentially inappropriate medications (PIMs) will be assessed within each clinic (cluster).

The two implementation strategies to be tested are (1) academic detailing (AD) and (2) AD plus the “Learn. Engage. Act. Process.” (LEAP) team-based quality improvement (QI) learning program (AD+LEAP). The first strategy, AD, is designed to provide *individual providers* with the knowledge and motivation to use the EBPs. The second implementation strategy, AD+LEAP, adds a *team*-based strategy that engages frontline providers and staff in incremental cycles of improvement in the use of the EBP with the support of a coach. The LEAP program provides coaching within a structured, paced curriculum over a 6-month period, during which teams complete one improvement project following a Plan-Do-Study-Act (PDSA) cycle of change. With both implementation strategies, participants will have access to a clinical dashboard or a similar resource, designed specifically for each EBP, that will provide actional data to inform improvement efforts. Clinical dashboards support providers in identifying high-risk patients and informing evaluation and treatment planning, and they are commonly used to increase uptake of EBPs in many areas of medicine [9–12] and throughout VHA [13–17]. They are scalable and sustainable and can form the core—but not the entirety—of a data-driven implementation program [18–20]; however, they are often used inconsistently in clinical practice [21].

### Evidence-based practices (EBPs)

The three EBPs will target (1) reducing inappropriate polypharmacy through proactive deprescribing in patients age 65 and older, (2) safe use of direct oral anticoagulants (DOACs), and (3) cognitive behavioral therapy as first-line treatment for insomnia.

Polypharmacy, often defined as the use of 5 or more medications, and hyper-polypharmacy (10 or more medications), are increasingly prevalent because of population aging and multi-morbidity [22–24]. Polypharmacy has been associated with increased risk of adverse drug events, drug-drug interactions, medication nonadherence, impaired functional status, cognitive impairment, and higher medical costs [25, 26]. Polypharmacy has, therefore, been a focus of quality improvement efforts, with a particular focus on older patients because of their greater susceptibility to medication harms. In recognition that polypharmacy may often be appropriate for patients with multiple comorbidities, the MIDAS EBP will focus on “inappropriate polypharmacy” and “potentially inappropriate medications” (PIMs) [27] in patients age 65 or older. PIMs are drugs that have an “unfavorable balance of benefits and harms compared with alternative treatment options;” guidance statements such as the American Geriatrics Society (AGS) Beers criteria [28] can aid in identifying patients using PIMs. The AGS Beers criteria is commonly used by clinicians, educators, and regulators and forms the basis for Healthcare Effectiveness Data and Information Set (HEDIS) quality measures related to medication management in older adults [28]. In the VA, a team of clinicians has developed an innovative practice known as VIONE to address inappropriate polypharmacy by encouraging and facilitating clinician review of each of a patients’ medications to determine whether each should be continued [29]; VIONE stands for “Vital, Important, Optional, Not indicated, and Every medication has a specific indication for use.” Components of the program include provider and pharmacist education about polypharmacy and VIONE when the program is initially implemented; access to multiple clinical dashboards that identify patients at increased risk for polypharmacy, as well as patients using specific drugs included in the AGS Beers criteria (the PIMs dashboard); a process for referring patients to pharmacists, who can perform medication reviews, using note templates in the electronic health record (EHR); and functionality to track medications that are discontinued, along with reasons for discontinuation. VIONE relies on a collaborative approach between providers and pharmacists to successfully deprescribe PIMs. VIONE has been recognized as a “gold status” practice by VHA leaders [30], is supported by the VA’s Academic Detailing Service, and has been implemented in over 100 clinical settings system-wide [31]. However, as highlighted by a recent evidence synthesis prepared for VA, there is a “glaring gap” in comparative effectiveness trials to identify the best approach to facilitate discontinuation of unnecessary and/or inappropriate medications [32].

The second EBP is the safe use of DOACs. DOACs are highly effective medicines to prevent harm from venous thromboembolisms, but when used inappropriately can also have severe side effects. In response, VA Pharmacy Benefits Management Services’ medication safety arm, the VA Center for Medication Safety (VA MedSAFE), has invested in national implementation efforts to promote safer practices for DOACs through medication use evaluations, national calls with pharmacists, traditional AD, and dashboards. In 2021, they also released an Anticoagulation Management Directive, which provides recommendations on best practices for quality assurance [33]. However, despite these efforts, safety concerns remain, and unsafe prescribing continues [34–38]. One particularly distinctive implementation effort is the DOAC Dashboard [36–38]. This tool has a series of flags for PIMs for every patient in VA. Unlike many dashboards, it is intended to be used at the point-of-care, primarily by anticoagulation pharmacists. Uptake of the dashboard has been rapid, with virtually every site in VA using it at least once per week.

Third, Cognitive Behavioral Therapy for Insomnia (CBTI) is recommended as first-line treatment for insomnia according to VA/Department of Defense (DoD) practice guidelines [39]. However, sedative hypnotic medications are still the most common treatment for insomnia, despite the associated risks of accidents, falls, and cognitive impairment [40–43]. The VA’s national evidence-based psychotherapy program has trained over 1000 therapists to deliver CBTI, yet programmatic and provider-level barriers (e.g., perceived priority) persist and limit adherence to treatment guidelines.

## Methods

Mixed methods analyses will be used to evaluate the following three aims:

1. *To compare the effectiveness of two implementation strategies (LEAP QI Learning Program + AD vs. AD alone)* on potentially inappropriate medication use, using a pooled analysis of effects across the three trials at 18 months, 2 years, and 3 years post-baseline at the clinic-level, based on monthly assessed data from 13–36 months*;*
2. *To compare the effectiveness of the two implementation strategies* on secondary outcomes specific to each trial at 18 months, 2 years, and 3 years post-baseline, based on monthly assessed data from 13 to 36 months; and
3. *To explore the effects* *of implementation, provider behaviors and experiences, and context*, on sustained improvements in potentially inappropriate medication use.

For the purposes of pooled analysis across the three trials, an analogous dichotomous outcome will be identified for each trial, reflecting the proportion of patients with potentially inappropriate medication use. This will effectively triple the number of clinics (8 clinics per trial; 24 total clinics) included in the analysis of Aim 1. Additionally, each trial will be analyzed as a standalone study. All three trials will have distinct secondary outcomes.

Our aims are designed to deepen commitment [44] to sustain EBP use by including measures that matter to different key constituencies including employees (e.g., workgroup functioning, job satisfaction), health system leaders (increased use of EBPs), and patients (e.g., reduction in PIMs) [6, 7, 45]. The combination of implementation strategies with measures that matter is designed to empower teams and individuals to increase meaning and purpose of their work, focused on the health and well-being of the Veterans we serve. Evaluation results will provide guidance as to which implementation strategy is more likely to lead to sustained outcomes.

### Human subjects protection

The MIDAS QUERI trials qualify as non-research conducted under the authority of Veterans Health Administration (VHA) operations, as it was designed and implemented for internal VHA purposes (to improve patient care) and not to produce information to expand the knowledge base of a scientific discipline.

In response to the designation of broad categories of activities as non-research in the *Federal Policy for the Protection of Human Subjects (Common Rule) in Title 38 Code of Federal Regulations Part 16 (38 CFR 16.102(l))* published January 19, 2017, the VHA enacted new policies and guidelines for determining non-research activities within VHA. In accordance with these VHA policies and guidelines, this program has documentation as non-research by Pharmacy Benefits Management, Office of Mental Health and Suicide Prevention, and Veterans Integrated Service Network (VISN) 10, which are each authorized to deem projects as non-research activities for which formal IRB oversight is not required, as defined per VHA Handbook 1058.05 in the section “Officials Authorized to Provide Documentation of VHA Program Office Non-Research Operations Activities” and later updated in section 5a of the VHA Program Guide 1200.21.

### Evaluation Design

This program is designed as a concurrent nested mixed methods evaluation [46, 47] in the context of cluster-randomized trials that will evaluate the effectiveness of AD+LEAP over AD as implementation approaches to improve the use of EBPs across three trials (See Additional file 1 for the SPIRIT checklist). Each trial will launch in quarterly increments over a 9-month period, each enrolling 8–12 clinics, randomized to one of the two implementation arms. For each trial, AD and AD+LEAP intervention activities will take place over a period of up to 12 months. Because our focus is on *sustainment* of improvements in clinical measures for each EBP, administrative data on key outcomes will be obtained over 36 months with a focus on comparisons at 18-month and 2- and 3-year post-randomization follow-ups.

### Partnered research

When research aims align with clinical priorities articulated by health system leaders, the likelihood of greater benefit can be dramatically amplified [48]. This program was developed in partnership with key offices within VHA, including Pharmacy Benefits Management, Office of Mental Health Services and Suicide Prevention (OMHSP), and executive leaders in two VISNs. We have worked closely with Pharmacy Benefits Management’s VA MedSAFE program and Academic Detailing Service. Our multi-faceted metrics are designed to deepen commitment to sustain the EBPs [44] and, in turn, to institutionalize them [45].

### Implementation strategies

As described above, all participating clinics will have access to regularly updated data from dashboards or similar resources. The dashboards provide clear, simple descriptions of care practices (e.g., patients at elevated risk of polypharmacy), thereby allowing easy identification of care variances to help detailers, individual providers, and clinic-level leadership identify opportunities for improvement [49]. In VHA, advances in medical informatics in design and content have produced increasingly user-friendly, responsive, and actionable dashboards, which have helped to amplify the work of clinicians and academic detailing pharmacists in invaluable ways. Our team is currently conducting a scoping review that will provide deeper knowledge of factors affecting uptake and effectiveness of dashboards [50]. Well-designed dashboards promote data-driven care optimization for individual care and population health management. All arms of care and outcomes of the trials will align with the clinical dashboards or a similar resource (e.g., the VIONE trial will rely on a VIONE practice dashboard and our measures will replicate those reported through the dashboard). Two implementation approaches that each use a dashboard or similar resource are described in the next sections (see Additional file 2 for StaRI Reporting Checklist details).

#### Academic detailing

Our AD intervention is modeled on existing AD principles. AD is a direct educational outreach of face-to-face (and more recently, virtual [51, 52]) interactions between academic detailers and clinicians that incorporate principles of adult learning theories, theory of planned behavior, and social marketing to improve the use of EBPs [53]. Using an accurate, up-to-date synthesis of the best clinical evidence in an engaging format, academic detailers ignite clinician behavior change, ultimately improving patient health. Evidence syntheses reveal that AD alone can be effective [54–57]; however, AD combined with other approaches (e.g., audit and feedback) is most effective in changing prescribing practices [58].

We will create an AD program that can be used and adapted for each intervention. The program will include a generalized approach based on existing recommendations, including documentation and training that will be general to the program and specific to each trial. For each trial, we will work with content experts and operational partners to develop 4–6 key messages that will be tightly linked with the primary outcome of the trial and the data in the respective clinical dashboard or resource. We will create detailer- and provider-focused educational materials to guide the detailer’s conversations with providers. This will include sample conversational scripts and tools to integrate detailing messages with provider-specific care patterns from the data.

Our detailers will be hired specifically for this project. They will attend training with the National Resource Center for Academic Detailing (NaRCAD) and through the VA Office of Academic Detailing. They will receive trial-specific education from each trial’s principal investigator and relevant content experts. They will shadow current detailers and will role-play the detailing sessions to practice conveying the key messages both internally and with non-participating practitioners. The sessions’ framing is based on the Theory of Planned Behavior [59, 60] and motivational interviewing [61].

The specific content of each visit will be tailored to address the specific context (barriers) identified at each participating clinic and for that specific provider. The detailer will start with an initial virtual visit with providers and other key staff at participating clinics; the detailer will meet with providers at each clinic for 15–30 min each. The detailer will review the dashboard in preparation for each visit to identify gaps and opportunities for improvement. A second, virtual visit will be completed four to eight weeks later to follow up with each participating provider.

For all three EBPs, we will identify provider-level barriers, including lack of knowledge about the EBP or uncertainty about the value of the practice [62]. Our AD strategy is designed to address these gaps by supporting individual providers in both use of the EBP and the use of clinical data to guide the practice.

Our AD approach will also include identifying a local champion prior to the first clinic visit. A “train-the-trainer” approach will be used to help ensure activities continue over the long term. The level and nature of champion engagement will be collaboratively determined by need and availability. Ideally, a local champion will shadow our detailer during visits and will be provided with training resources and coaching. Our detailer will develop a plan with the local champion to continue to reach out to new providers as appropriate to more deeply embed and sustain the practice and to track EBP use with the relevant data resource.

#### The Learn. Engage. Act. Process. (LEAP) program

Through prior work, we have identified common barriers encountered when implementing EBPs. This work, guided by the Consolidated Framework for Implementation Research (CFIR), has repeatedly identified a lack of planning, not consistently engaging key stakeholders, and not taking time for reflecting and evaluating on progress and impact in EBP implementation efforts [63–65]. LEAP, a blended implementation approach [66], is specifically designed to address these barriers by interweaving four discrete, evidence-based implementation strategies: (1) create a learning collaborative, (2) assess for readiness and identify barriers and facilitators, (3) audit and provide feedback, and (4) conduct cyclical small tests of change [67, 68].

**Fig. 1:**
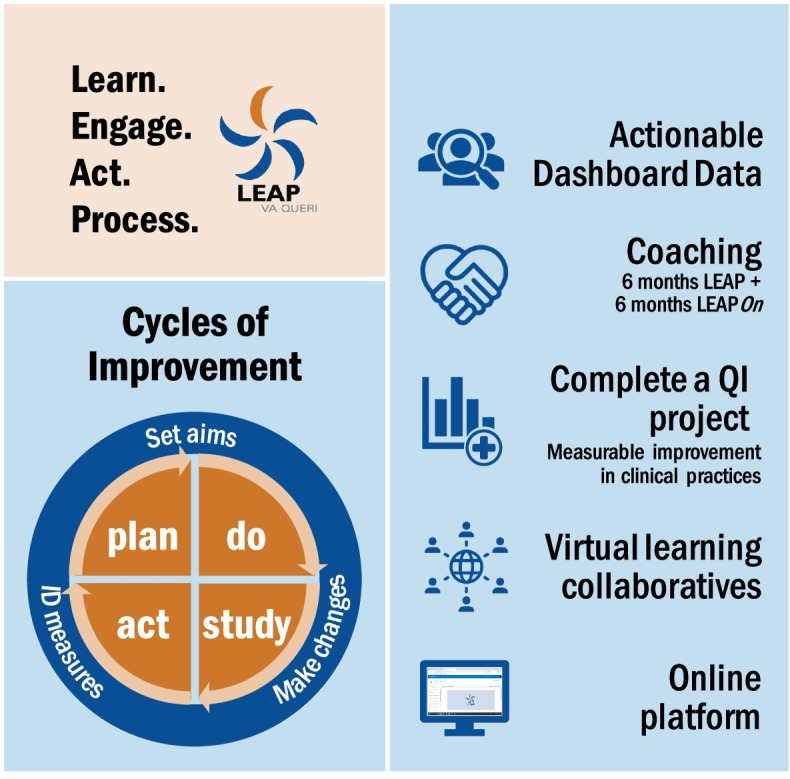
LEAP program components

The LEAP QI program engages frontline teams in sustained incremental improvements of EBPs over a 6-month period of hands-on learning, designed for busy clinicians as listed in Fig. 1. The Institute for Healthcare Improvement’s (IHI) Model for Improvement and PDSA cycles of change provides the core foundational approach [58] for team-based, hands-on learning, and coaching support with a QI network to enhance learning and accountability.

The LEAP curriculum was adapted from a Massive-Open Online Course (MOOC) developed by HarvardX in collaboration with IHI [69]. Materials from the MOOC were adapted for LEAP by (1) designing for teams rather than individuals, (2) streamlining materials to accommodate busy frontline clinicians, and (3) lengthening program duration to provide more time to complete an improvement project. The LEAP curriculum includes brief videos, short readings, and easy-to-understand templates and tools, using selected content developed by IHI and HarvardX. The curriculum is paced, with new guidance released on a weekly basis through an online platform (SharePoint Online). Assignments completed in LEAP (i.e., project charter) can be drawn on for continued future improvement efforts. Continuing education (CEs) are available through VA’s Talent Management System (TMS).

Each clinic participating in LEAP forms a QI team. In our cluster randomized design (described below), teams will participate in cohorts of 4-6 to create a learning collaborative. LEAP coaches interact with teams in individual webinar sessions in the early weeks of LEAP and later via virtual collaboratives with all teams. LEAP teams choose aims, plan projects, and monitor data to bring about meaningful changes based on the specific needs surrounding the EBP at hand. The LEAP implementation strategy also includes a 6-month maintenance component, called LEAPOn, that provides monthly collaboratives for teams to encourage continued work on PDSA cycles.

So far, 49 teams have completed LEAP, comprising 276 frontline staff, clinicians, and Veterans. Based on first-year results, LEAP measurably increased confidence in using QI methods, and participants were satisfied or very satisfied (81-89%) with all LEAP components [70]. In addition, 96% agreed or strongly agreed that LEAP was relevant to the needs of their program. Post-LEAP, teams intended to continue to optimize care for their patients; however, participants struggled most with the lack of available time for QI amid competing clinical priorities.

### Conceptual framework for evaluation

MIDAS QUERI focuses specifically on the sustained use of EBPs. The Dynamic Sustainability Framework (DSF) asserts that “[o]ngoing quality improvement of interventions is the ultimate aim…[because] evidence solely from clinical trials [is insufficient] and…quality improvement processes focused on intervention optimization are ultimately more relevant to achieve sustainment.” [71] Sustainment science literature [7, 45] and other implementation science frameworks [72, 73] all affirm the necessity of ongoing optimization. Thus, at the center of the DSF is the need to engage individuals and teams in continual adaptation and optimization through learning cycles like the PDSA cycles foundational in QI [72]. However, clinical teams have significant challenges doing PDSA cycles because of patient care demands and they must navigate constant changes in infrastructure, policies and procedures, and staffing, all of which leave little time for implementing improvements. Nevertheless, if frontline teams do not invest time and effort into making improvements, change will not happen and/or will not be sustained, leading to widespread failures across the system. The LEAP and AD strategies are specifically designed to engage busy frontline employees in continuing incremental optimization of each EBP.

**Fig. 2:**
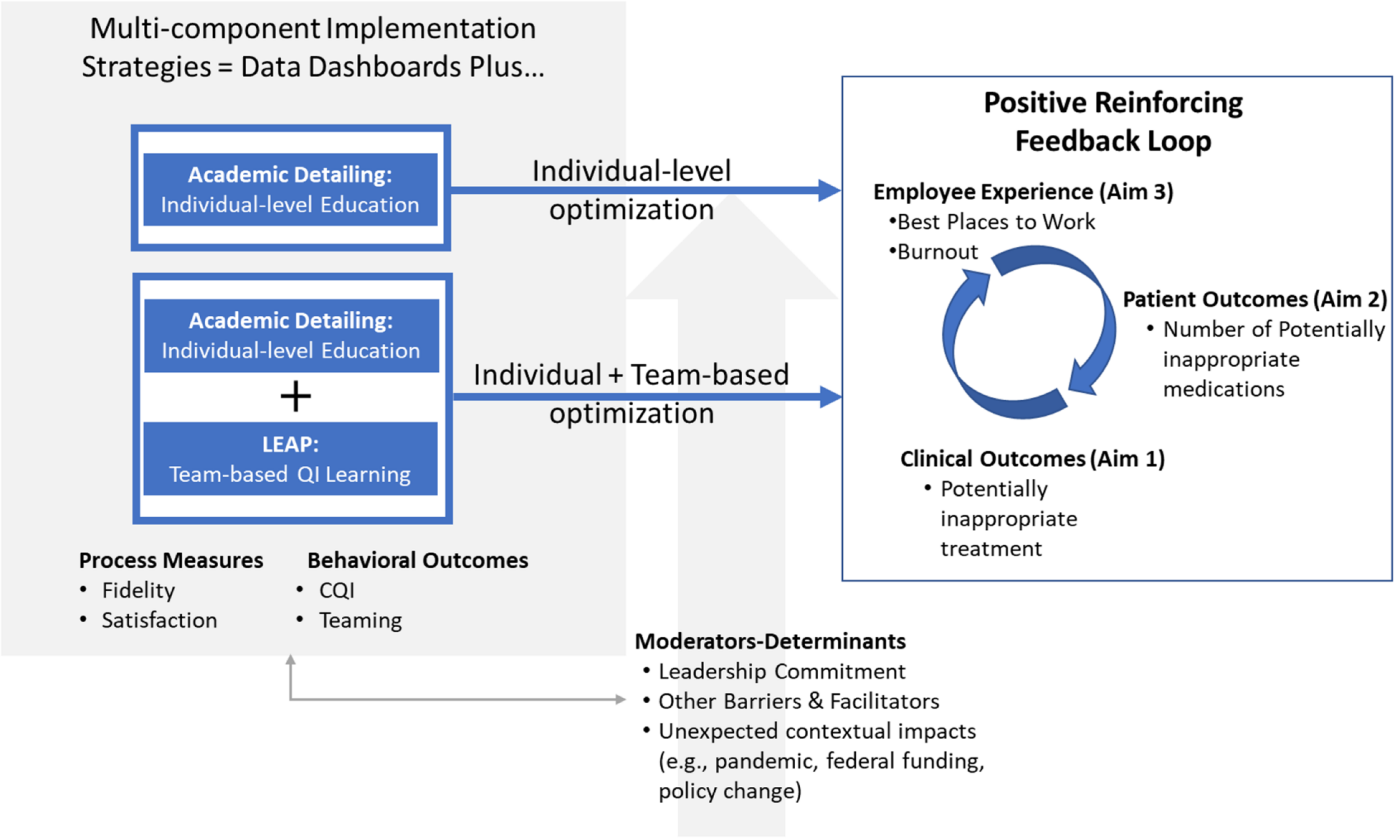
MIDAS conceptual framework

Sustainability research highlights the need to identify outcomes important to multiple stakeholders for change to be fully integrated as routine care [7, 45]. Figure 2 shows our conceptual framework. At the heart, is a positive reinforcing feedback loop between three categories of outcomes, each designed to meet the needs of three key constituencies: (1) employees who deliver treatments; (2) health system leaders; and (3) patients. Our strategies are designed to move individuals and/or teams into a virtuous cycle where engaging in optimization brings visible improvements in work-life (e.g., burnout, satisfaction as measured by “Best Places to Work”) as employees are motivated [74, 75] by seeing measurable improvements in near-term service outcomes that matter to clinical leaders (increased use of EBPs) and patients who experience improved clinical outcomes. Sustained change relies on building ever-stronger coalitions of support that can occur when outcomes are visible and communicated widely. This increased visibility with supervisors and other clinical leaders will help to foster willingness to allow the space and time needed to engage in optimization [4, 7, 8, 45]. Increasing capacity for change, especially through teamwork, is strongly associated with lower burnout among clinicians [76]. We will combine qualitative findings with quantitative measures to help explain changes (or lack thereof) over time. Our AD strategy is based on Theory of Planned Behavior, where attitudes, subjective norms, and perceived behavioral control shape behavioral intentions that lead to engaging in cycles of optimization of personal work processes. The LEAP strategy relies on teaming theory [77] and engagement in continuous QI [78] and provides team-based structured coaching as teams learn to plan and execute PDSA optimization cycles.

The effectiveness of our implementation strategies will be moderated by contextual determinants (i.e., barriers and facilitators) influencing teams’ and individuals’ ability to engage in optimization. These contextual determinants will be assessed using a newly developed pragmatic Context Assessment Tool (pCAT; unpublished) that assesses nine constructs across three of the domains of the CFIR (Innovation Characteristics, Outer Setting, and Inner Setting). This prioritized list of constructs was chosen based a series of context assessments during implementation evaluations in VHA [63–65]. The COVID-19 pandemic has heightened the awareness of how other unexpected impacts that may also influence this pathway to the positive reinforcing feedback that is designed to keep individuals and teams engaged in optimization.

### Clinic selection and eligibility

We will work with our operational partners to identify candidate clinics that want to reduce their use of PIMs based on the topic for each respective trial. We will provide an orientation to the topic, introduction of the dashboard, and overview of the two implementation intervention arms. Prior to implementation, we will work with interested clinics to ensure they have met the preconditions necessary to begin *sustained* optimization of the EBP: (1) a team leader or champion; (2) an identified department with service leadership buy-in and control over the processes/practices impacted by the implementation; (3) readily accessible data to monitor process and impact of the implementation and use of the EBP, e.g., through an easy-to-access dashboard; and (4) installment of key components needed to support the EBP (e.g., installation of a specific note template in the EHR system). We will recruit four to six clinics per arm per trial; a clinical leader will provide assent to participate and enroll.

### Clinic randomization

Within each trial, clinics will be randomized after assenting to participate (equivalent to enrollment). Clinics will be assigned to one of two arms by a statistician, stratified further by clinic type (medical center, community clinic, or Community Living Center) if needed to ensure partial balance between arms with respect to potential confounders associated with culture and complexities associated with clinic location [79, 80].

### Outcomes and analyses

As part of a pooled analysis, we will compare the same two implementation strategies across all three EBPs and take a unified approach to implementation and evaluation across the trials. Table 1 shows MIDAS measures, data collection timeframe, and data sources. While a unified dichotomous outcome, i.e., PIMs, was identified for each trial to allow for the pooled analysis, each trial will also be analyzed individually (see Table 1).

**Table 1:** MIDAS measures showing data sources and timepoints by aim

Aim	Type	Source
Aim 1: Primary outcome		
**Service outcomes**		
Proportion of potentially inappropriate medications	Quantitative	CDW administrative data
Aim 2: Secondary outcomes		
**Service outcomes**		
VIONE trial		
Potentially inappropriate use of proton pump inhibitors (PPIs), aspirin, and central nervous system (CNS) active medications (e.g., muscle relaxants), analyzed one at a time	Quantitative	CDW administrative data
Monthly medication costs for all drugs	Quantitative	CDW administrative data
Number of pharmacist medication reviews	Quantitative	CDW administrative data
DOAC trial		
Dashboard flags including potential mis-dosing, potential medication interactions, or concern for nonadherence	Quantitative	CDW administrative data
CBTI Trial		
Prevalence of any CBTI receipt	Quantitative	CBTI note templates completed by CBTI therapists
Mean CBTI sessions completed	Quantitative	CBTI note templates completed by CBTI therapists
Referrals to CBTI among primary care patients actively following with the clinic who are not in hospice/palliative care	Quantitative	Consult requests in the medical record or by monthly therapist reports
**Patient outcomes**		
VIONE trial		
Number of inappropriate medications by patient		CDW administrative data
Aim 3: Exploratory outcomes		
**Employee behavioral outcomes**		
Continuous quality improvement assessment	Quantitative	LEAP and AD participants
Workgroup cohesion and engagement scale	Quantitative	LEAP and AD participants
QI skill application	Quantitative	LEAP participants
**Employee experience**		
Burnout	Quantitative	LEAP and AD participants
Best places to work	Quantitative	LEAP and AD participants
**Process evaluation**		
Pragmatic Context Assessment Tool	Qualitative/quantitative	LEAP participants
Semi-structured interviews	Qualitative	Purposive sample of LEAP and AD participants
How often the provider uses the dashboard	Quantitative	CDW administrative data
Rates of new DOAC starts compared to warfarin starts	Quantitative	CDW administrative data
Fidelity	Qualitative/quantitative	Academic Detailer and Champion/LEAP Coaches
Provider satisfaction	Qualitative/quantitative	LEAP and AD participants
Intentions	Qualitative/quantitative	LEAP participants
Detailing visit documentation	Qualitative/quantitative	Academic detailer and champion
Coaching documentation	Qualitative/quantitative	LEAP Coaches
Semi-structured interviews	Qualitative	Purposive sample of LEAP and AD participants

#### Aim 1: primary outcomes and pooled analysis

Although each trial will be conducted as an independent study, our primary aim is to compare across trials the effectiveness between the two implementation strategy arms in reducing PIMs during post-implementation period. To this end, we defined a unified primary outcome to allow us to combine the results *across* the three trials. The unified primary outcome will be operationalized based on a patient-level dichotomous response indicating PIM use (yes/no) among patients at-risk of PIMs, i.e., among those who may benefit from the specific EBP each month. The monthly patient-level PIM use response will be summarized to clinic-level month-by-month percentage of potentially inappropriate use using administrative data from baseline to 36 months, with months 13–36 as the post-implementation follow-up period. Each trial-specific monthly data will be cross-sectional, i.e., different patients may be included in each month.

For *inappropriate polypharmacy*, the clinic-month outcome will be the proportion of patients who had medication possession (based on VA pharmacy fill data) of one or more medications from the AGS Beers criteria that are included on the VIONE PIMs dashboard [28] (numerator) among patients age 65 or older, not receiving palliative care, and followed by the clinic (denominator). For each drug included on the PIMs dashboard, there are associated business rules that define when medication use is flagged as potentially inappropriate; these same criteria will be applied in this trial. For example, the use of a first- or second-generation anti-psychotic drug is flagged as potentially inappropriate unless there is a diagnosis of schizophrenia or bipolar disorder. These criteria had previously been determined by VIONE’s Subject Matter Expert group, which provides VIONE with guidance on translating deprescribing criteria into the most practical and appropriate rules for use on the dashboard. Altogether, the following AGS Beers medications from the PIMs dashboard will be included in the analysis: anticholinergics, antipsychotics, aspirin, benzodiazepines, long-acting sulfonylureas, muscle relaxants, non-steroidal anti-inflammatory drugs (NSAIDs), proton pump inhibitors (PPIs), sliding scale insulin, and Z-drugs.

For *DOAC safety*, the outcome will be the proportion of patients with potentially inappropriate prescribing out of those using DOACs, as measured by “flags” (e.g., potential mis-dosing based on renal function and other indicators) on the DOAC dashboard. The DOAC flagging system is based on Food and Drug Administration (FDA) indications and has been in clinical use since 2018. Components of the outcome include inappropriate dosing for the given indication and the use of DOACs in contraindicated settings (such as valve replacements).

For *first-line treatment for insomnia*, the outcome will be the proportion of patients with a new prescription for a sedative-hypnotic medication who have not had CBTI in the prior 12 months out of all primary care patients actively following with the clinic and are not in hospice/palliative care.

For all three trials, medication use (yes/no) and possession of active prescription for each month will be determined using exposure days based on supply days, and use will be determined by the exposure status on day 1 of each month. We will also do sensitivity analyses based on the criteria of use anytime during the month as well as PIMs defined to medications used chronically, for example, greater than 90 of the 180 prior days.

For each trial we will first compare demographic characteristics (age, sex, and race) of patients at risk of PIMs in the first month of implementation between the two arms. We will then obtain, for each trial by arm, crude monthly percentages (along with the corresponding 95% confidence intervals) of PIMs, averaged across clinics randomized to each arm and weighted by clinic-month size. For each trial, we will plot the monthly clinic level percentages over the follow-up 13–36 months to graphically assess if the difference between the two arms can be meaningfully summarized across the three trials with the unified outcome. If we find, for example, that trends between-arms over post-implementation months differ notably across the three trials, unified results comparing AD+LEAP vs. AD arm across trials may not be meaningful, and we will only conduct analyses separately by each trial.

For comparison between arms, we will use generalized estimating equations (GEE) with clinic-level monthly percent of PIMs among patients at risk during post-implementation period (months 13 to 36) as the dependent variable. The model will include indicators of two trials with one trial as the referent category to account for differing underlying levels of inappropriate medication use across trials. The model will also include follow-up time in months and the LEAP+AD arm indicator with AD as the referent category and will adjust for serial correlation within clinic over time. We will also include time by arm interaction to assess if the magnitude of the difference between LEAP+AD vs. AD changes over time. If the interaction is significant, we will estimate between-arm difference at 18 months as well as at 2- and 3-years separately based on the model with the interaction term. On the other hand, if the interaction is not significant, this would indicate between-arm difference not to differ at the three follow-up times of interest (18, 24, and 36 months), and thus we will drop the interaction term and the parameter estimate of the LEAP+AD arm indicator will be used to estimate the time-averaged difference in percentage of patients with inappropriate medications during the post-implementation period in clinics randomized to LEAP+AD compared to clinics randomized to AD.

If we find notable baseline demographic differences between arms within trials, we will use a generalized linear mixed model (GLMM) with logit link to estimate the between-arm difference while adjusting for baseline age, sex, and race difference with monthly person-level response (yes/no) data from the post-implementation period of months 13 to 36. In addition to time, AD+LEAP indicator, and trial type indicators as predictors, the GLMM model using patient-level data will include patient age, sex, race, and random intercepts for patients nested within clinic to adjust for potential correlation within clinics and serial correlation over time. The parameter estimate for the LEAP+AD arm indicator will be used to estimate the time-averaged odds of inappropriate medication use during the post-implementation period for patients in clinics randomized to LEAP+AD compared to the odds of the same patients if their clinics were randomized to AD. Although the GEE and GLMM models give different summary estimates with different interpretations, the GLMM model allows for adjusting for patient characteristics, and a consistent substantive conclusion will assure us of the evidence for the effect of LEAP when added to AD. Similar to the GEE model, we will test if the odds ratio of LEAP+AD vs. AD changes over time by including time by arm interaction term, and if the interaction term is significant, we will obtain adjusted odds ratios associated with LEAP+AD compared to AD at 18 months, 2 years, and 3 years.

For each trial, we will also compare AD and AD+LEAP to usual care controls. To do this, we will perform a non-randomized secondary analysis for each trial. The analysis will have the same primary outcome variable and use the same generalized linear mixed model (GLMM) with logit link. The primary control group will be all non-participating sites. We will also use a secondary analysis, where for each intervention site we will have two control sites that are matched on clinic size (within 50%), pre-intervention outcome rate (within 30 rankings of all sites), and region of the country. These analyses will adjust for the clinic-level variables clinic size, intervention outcome rate, region of the country, and the patient-level variables age, sex, and race.

#### Aim 2: secondary outcomes and analyses

Secondary outcomes for VIONE will be the prevalence of potentially inappropriate use of PPIs; the prevalence of potentially inappropriate use of aspirin; and the prevalence of potentially inappropriate use of central nervous system (CNS) active medications (muscle relaxants, anti-psychotics, Z-drugs, and benzodiazepines) or anticholinergic drugs; number of inappropriate medications at a patient level; monthly medication costs for all drugs, without regard to appropriateness; and number of pharmacist medication reviews.

Secondary outcomes for the DOAC trial will be the sub-components of the “flags” on the dashboard. These include potential mis-dosing, potential medication interactions, or concern for nonadherence. This follows the organizational structure of both the presentation of the flags on the dashboard and the key messages provided to the AD and LEAP teams. Process outcomes will be how often the provider uses the dashboard and rates of new DOAC starts compared to warfarin starts. These outcomes will be kept in alignment with our other work using the dashboard [37].

In stand-alone analyses of the CBTI trial, the primary outcome will be the prevalence of any CBTI receipt among primary care patients actively following with the clinic who are not in hospice/palliative care. Secondary outcomes will be the mean CBTI sessions completed and referrals to CBTI. Receipt of any CBTI and mean number of sessions will be measured by extracting from the medical records' CBTI note templates completed by CBTI therapists. CBTI referrals will be measured according to consult requests in the medical record or by monthly therapist reports.

Analyses of secondary outcomes such as percent of potentially inappropriate use of PPI or mean number of CBTI sessions at each clinic month will be similar to that of the primary outcome using the GEE model accounting for correlation over time. We will also conduct separate analyses by trial with the dependent variables that are unique to each trial. For example, for the polypharmacy trial, the secondary outcome of interest is count of medications flagged as inappropriate based on Beers’ criteria [28]. We will compare monthly rates of Beers’ list medication use between implementation strategies using GLMMs with log link.

#### Aim 3: exploration of potential predictors of clinical outcomes

Our process evaluation will follow a multi-phase concurrent nested mixed methods design [81]. This design has three purposes: (1) help prepare all stakeholders and participants prior to the start of each trial; (2) monitor the progress of implementation; and (3) explain summative findings. Overall priority is placed on quantitative methods that guide the trials, while qualitative methods are embedded or “nested” within conduct of the trials.

Employee behavior and experience measures will be collected via five scales as listed in Table 1. Surveys will be administered via online link within invitation emails; administration will occur at baseline and 18 months post-baseline; satisfaction will be elicited at the end of each intervention (upon completion of the 6-month “core” LEAP program for LEAP team members and at the end of each AD visit for AD participants). Descriptive statistics will be generated and tests for differences across implementation strategy arms will be conducted using mixed models to account for within-clinic correlation.

Qualitative data will be collected prior to and 18 months following baseline via semi-structured interviews (virtual by telephone or conferencing software (e.g., MS Teams platform)). A purposive sample of key people (clinic leaders, supervisors, providers, and staff) at each clinic will be invited to participate so we can better understand the context in which the implementation strategies are/were deployed. The interview guides and qualitative analyses will be guided by the CFIR to identify potential and actual barriers and facilitators [63–65]. Principles embedded within the DSF will guide exploration of the degree of engagement in QI and teamwork [71]. Prior to implementation, this information will help inform the work of the academic detailers and LEAP coaches; post-implementation, this information will help to explain quantitative findings within and across the trials. Interviews will be audio-recorded and transcribed verbatim. Pre-implementation, interviews will focus on collecting practical information using a rapid analysis approach [82, 83] to help tailor and adapt implementation for each participating clinic (see Additional file 3 for master interview guide). Post-implementation, qualitative analyses will seek insights on what kinds of improvements were made, barriers and facilitators to making improvements, reflections on/satisfaction with participation in AD/LEAP, and explore *relationships* between determinants, participants, and key stakeholders and how these may lead to building coalitions of support [7, 8]. We will combine qualitative findings with quantitative measures from Aims 1 and 3 to help explain changes (or lack of) over time.

Our process evaluation will rely on quantitative and qualitative data sources. Fidelity to each implementation strategy will be tracked by interventionists (the detailers and LEAP coaches) completing a mixed-methods self-assessment tool after each interaction (a coaching session for LEAP, detailing contact for AD). These assessments will be used to guide coach-supervisor and peer reflections on improvements, problem-solving, and mitigating barriers and amplifying facilitators of improvement efforts. We will also track participation by participants (individuals scheduled for detailing and/or LEAP team members) and completed assignments by LEAP teams. The academic detailer and LEAP coaches will enter notes for each interaction into a tracking system for each strategy. This data will be combined with pre-implementation and 18-month semi-structured interview data for further insights into barriers, facilitators, and problem-solving approaches used by LEAP coaches and detailers. Quantitative and qualitative data will be combined at the analysis or interpretation phase.

#### Economic evaluation

We will use a micro-costing method [84, 85] to determine the costs to deliver LEAP and AD. The LEAP coaches and academic detailers developed a list of the activities they will perform for each participating site. Depending on the specific activity, they determined the best way to record the time spent on each activity—e.g., logging the start and stop time each time the activity takes place vs. setting an estimated average time for activities that take approximately the same amount of time for each incidence (such as recurring meetings, responding to quick queries via e-mail, meeting preparation, etc.). In the latter case, the coaches and detailers simply record the occurrence of the activity, which is then assigned the estimated time. The coaches and detailers will log times for each activity, categorized by participating site, in a time tracking database. Using data from this database, we will calculate the average time required for each activity and apply this to the number of times it takes place over the course of performing the implementation strategy at a site. These data can then be used to determine an estimated total time required to perform the implementation strategy (LEAP or AD) at a site, which, combined with the hourly cost of the LEAP coach or detailer, can be used to calculate the total cost of employing the strategy at a site.

## Discussion

The MIDAS program of QI trials is unique and ambitious in unifying conduct of three cluster randomized trials on three EBPs across diverse settings (medical centers and community-based clinics) and within different clinical specialties (clinical pharmacy, primary care, and mental health) within VHA. Implementation strategies were designed based on previous implementation studies (LEAP) and in partnership with VHA operations leaders (AD). Two-arm trials to compare AD alone or adding LEAP are combined with multi-phase concurrent nested mixed methods process evaluations to ensure valued and much-needed learning, regardless of trial outcomes [81].

Our two implementation strategies each target a different level: AD intervenes with individuals to build capability and motivation for optimizing personal work processes while LEAP intervenes with teams to build capability and motivation for optimizing broader clinical processes. Using both may provide a “winning” multi-level strategy where one builds on the strengths of the other for lasting change.

Our overarching goal is sustainability. Our approaches and measures draw on sustainability science that point to the need to engage clinicians and staff in ongoing optimization or QI. Engaging frontline teams and individuals in continued practice optimization can often feel like an up-hill battle. The “gravity” we have encountered in our past work [70]—and reinforced in findings by others—is a lack of time [86–89]. On the other hand, when mission and values translate into aligned priorities up and down the system, it is motivational—it is thrilling—to work within communities—teams and coalitions within organizations—and to be a part of something larger [87, 88]: serving Veterans and making a difference. There is no silver bullet or magic solution to moving individuals or teams into this space but our approach to making small changes that are feasible to do within demanding clinical settings, has the potential to coalesce forces for large-scale positive impact [7]. By increasing the visibility of learnings and successes, one individual/one team can make a positive impact. We aim to help shift power to these agents for change by focusing on small doable, incremental changes that add up to significant impact over time. Especially because of forces outside the control of our strategies, our multi-phase mixed methods evaluation approach is essential. The combination of qualitative data informing or explaining quantitative findings will help ensure we generate learnings and insights that will benefit all stakeholders.

This QI program has limitations. Our primary outcomes are at 18 months and 2 and 3 years post-baseline. Significant secular impacts are increasingly common (e.g., pandemic, flood, fire) and the causal pathway is not clear between intervention and outcomes. One certain disruption is VHA’s migration to a new EHR system planned during the trial period, which may impact the availability and reliability of administrative data and may impact clinics’ and individuals’ ability to engage fully in optimization as they wrestle with learning a new way of working. However, we have built-in multiple dimensions of measures (employee-focused, system-focused, and patient-focused) along with qualitative and process data that are designed to help tailor support and monitor and explain findings that can reveal important insights such as which settings resulted in the highest impact with which combination of implementation strategies; we have also included a secondary analysis with matched controls to factor out systemwide disruptions or trends.

The landscape is fast-moving with respect to intervention options. For example, for CBTI, computer-based CBTI, group-based treatment, and a briefer version of the full CBTI model are all quickly building evidence and are appealing in their ability to ease pressures with a limited number of providers trained in CBTI. VHA leaders are also paying increasingly closer attention to the need for CBTI and may implement new policies to motivate its use as a first-line treatment for insomnia, e.g., adding alerts that recommend CBTI each time a provider tries to order a sleep medication. Each of these approaches may have their own champions at various levels within the organization and with their own preferences and partners for implementation. Thus, our team will remain open to the best approach for as long as possible before launching that trial. This reality highlights how important our ability to remain agile and responsive is, and as we strengthen our partner relationships for successful trial conduct with in-depth evaluation. As embedded researchers engaged in systemwide QI, we recognize the need to align with system priorities [90] and be attuned to findings that will best improve care for patients [91].

## Supplementary Information

**Additional file 1.**

**Additional file 2.**

**Additional file 3.**

## Data Availability

Data will be available upon request after completion of the study.

## Abbreviations

AD: Academic detailing
AGS: American Geriatrics Society
CBTI: Cognitive Behavioral Therapy for Insomnia
CE: Continuing education
CFIR: Consolidated Framework for Implementation Research
CNS: Central nervous system
DoD: Department of Defense
DOACs: direct oral anticoagulants
DSF: Dynamic Sustainability Framework
EBPs: Evidence-based practices
EHR: Electronic health record
FDA: Food and Drug Administration
GEE: Generalized estimating equations
GLMM: Generalized linear mixed model
HEDIS: Healthcare Effectiveness Data and Information Set
IHI: Institute for Healthcare Improvement
LEAP: Learn. Engage. Act. Process.
MIDAS: Maintaining Implementation through Dynamic Adaptations
MOOC: Massive-Open Online Course
NaRCAD: National Resource Center for Academic Detailing
NSAID: Non-steroidal anti-inflammatory drugs
OMHSP: Office of Mental Health Services and Suicide Prevention
pCAT: pragmatic Context Assessment Tool
PDSA: Plan-Do-Study-Act
PIM: Potentially inappropriate medication
PPI: proton pump inhibitor
QUERI: Quality Enhancement Research Initiative
QI: Quality improvement
TMS: Talent Management System
VA: Veterans Affairs
VA MedSAFE: VA Center for Medication Safety
VHA: Veteran Health Administration
VIONE: Vital, Important, Optional, Not indicated, and Every medication has a specific indication for use
VISN: Veterans Integrated Services Network
