# Supplementary material for: Maintaining Implementation through Dynamic Adaptations (MIDAS): protocol for a cluster-randomized trial of implementation strategies to optimize and sustain use of evidence-based practices in Veteran Health Administration (VHA) patients
Source: Implement Sci Commun. 2022 May 14;3:53. doi: 10.1186/s43058-022-00297-z (PMC9107220; doi:10.1186/s43058-022-00297-z)
Supplement: Supplementary file 3 — Additional file 3. [file 43058_2022_297_MOESM3_ESM.docx]

**Additional File 3**

**MIDAS Master Interview Guide**

My name is [interviewer name] and I am from the VA in ___________. With me are [2^nd^ interviewer name] and [note taker name]. We are part of the MIDAS QUERI team made up of researchers and physicians based at the Center for Clinical Management Research at the Ann Arbor VA. MIDAS is a quality improvement program focused on increasing appropriate prescribing of [DOACs/VIONE/CBTI] by decreasing the variability in implementation of evidence-based practice. We’re interested in hearing your thoughts about barriers and facilitators to appropriate prescribing of [DOACs/VIONE/CBTI].

Taking part in this interview is completely voluntary. Anonymity and confidentiality of participants will be preserved by limiting access to identifiable information to only our project team. Findings shared outside of our team will be de-identified and reported in aggregate at the facility level.

The interview will last no longer than 1 hour. You can skip any questions you prefer not to answer. You can stop the interview at any time. We would like to record this interview so that we have a complete and accurate record of the information you provide. You may ask to pause or stop recording at any time. The audio file will be stored in a restricted access file on a secure VA server with access limited to a select set of team members.

Do I have your permission to record the interview?

Do you have any questions before we get started?

[Turn on recorder] Okay, to confirm, I’m starting the recording. Is this ok with you?

1. Please tell me your title and role in VA.
   1. How long have you been in this position?
   2. How long have you worked at this facility? (Where did you work prior?)
2. Do you use VIONE/CBTI? Why or why not?
3. Can you describe your [DOAC/VIONE/CBTI] management process? How do you fit it into your workflow?
   1. Explore the role and degree of integration of the dashboard/other tools and resources.
   2. How has this process changed? Why? (e.g. root cause analysis, sentinel event, quality improvement)
4. What do you feel are the barriers and facilitators to appropriately prescribing [DOACs/VIONE/CBTI]?
   1. How do you find out about new clinical processes/approaches/evidence?
5. How important is optimization of [DOACs/VIONE/CBTI] compared to other priorities?
   1. How important do you think optimization of [DOACs/VIONE/CBTI] is to your patients?
6. What has your experience with academic detailing been? [KNOWLEDGE OF THE INTERVENTION]
   1. Quality/Process Improvement?
   2. Lean?
7. What are your recommendations for how to better manage [DOACs/VIONE/CBTI]? [INTERVENTION CHARACTERISTICS – EVIDENCE STRENGTH & QUALITY]
   1. How could people in different roles (e.g., nurses, medical assistants, pharmacists, administrative, etc.) help better manage [DOACs/VIONE/CBTI]?
   2. How do you feel academic detailing might help?
   3. How do you feel about a detailer who is a non-physician/pharmacist? [ATTITUDES & BELIEFS]
   4. What organizational changes would make it easier to use these tools?
8. To what extent do you participate in improving management of [DOAC/VIONE/CBTI]?
   1. Can you describe specific barriers/facilitators?
9. Is there anything I haven’t asked about today that you feel is important to mention with regards to appropriate prescribing of [DOACs/VIONE/CBTI]?

Thank you very much for taking the time to participate in this interview. Your thoughts and insights have been immensely helpful and will be incorporated into our work as we continue to focus on decreasing variability in the implementation of evidence-based practices designed to facilitate appropriate prescribing of [DOACs/VIONE/CBTI].
